# Why fly the extra mile? Latitudinal trend in migratory fuel deposition rate as driver of trans‐equatorial long‐distance migration

**DOI:** 10.1002/ece3.2388

**Published:** 2016-08-25

**Authors:** Yaara Aharon‐Rotman, Ken Gosbell, Clive Minton, Marcel Klaassen

**Affiliations:** ^1^ Centre for Integrative EcologySchool of Life and Environmental ScienceDeakin University75 Pigdons RdGeelongVictoriaAustralia; ^2^ Australian Wader Studies Group1/19 Baldwin RoadBlackburnVictoria3130Australia

**Keywords:** Body stores, geographic variation, optimal migration strategy, tracking, waders

## Abstract

Trans‐equatorial long‐distance migrations of high‐latitude breeding animals have been attributed to narrow ecological niche widths. We suggest an alternative hypothesis postulating that trans‐equatorial migrations result from a possible increase in the rate at which body stores to fuel migration are deposited with absolute latitude; that is, longer, migrations away from the breeding grounds surpassing the equator may actually enhance fueling rates on the nonbreeding grounds and therewith the chance of a successful, speedy and timely migration back to the breeding grounds. To this end, we first sought to confirm the existence of a latitudinal trend in fuel deposition rate in a global data set of free‐living migratory shorebirds and investigated the potential factors causing this trend. We next tested two predictions on how this trend is expected to impact the migratory itineraries on northward migration under the time‐minimization hypothesis, using 56 tracks of high‐latitude breeding shorebirds migrating along the East Asian‐Australasian Flyway. We found a strong positive effect of latitude on fuel deposition rate, which most likely relates to latitudinal variations in primary productivity and available daily foraging time. We next confirmed the resulting predictions that (1) when flying from a stopover site toward the equator, migrants use long jumps that will take them to an equivalent or higher latitude at the opposite hemisphere; and (2) that from here onward, migrants will use small steps, basically fueling only enough to make it to the next suitable staging site. These findings may explain why migrants migrate “the extra mile” across the equator during the nonbreeding season in search of better fueling conditions, ultimately providing secure and fast return migrations to the breeding grounds in the opposite hemisphere.

## Introduction

Migration has evolved as an adaptation toward avoiding risks, such as unfavorable thermal conditions, food shortage, predation, and disease, as well as seizing opportunities where and when they arise (e.g., Salewski and Bruderer 2007; Louchart 2008). For many high‐latitude breeders, notably cold and food shortage are important drivers of movements toward the equator before the winter sets in (Newton 2003). Even if wintering at high latitude is still an option, the advantages in thermostatic cost close to the tropics may outweigh the extra costs of flight (Wiersma and Piersma 1994). However, these explanations for the seasonal long‐distance migration from high latitudes fail to explain long‐distance trans‐equatorial migrations as found in many animal taxa which breed in the Northern Hemisphere and winter in the Southern Hemisphere. The prime reason suggested for these extra‐long, trans‐equatorial migrations is that migrants have narrow ecological niche widths, using equivalent habitats (to which they are specialized) at both sides of the equator during the most productive season (Newton 2003). But in some cases, such as in many Arctic breeding, long‐distance migratory shorebirds, apparently suitable and similar habitats, are passed, or temporarily used and then left, for more southern destinations. We here build a case that, possibly counterintuitively, these long trans‐equatorial flights actually enhance the chances of a successful, rapid, and timely migration back to the breeding grounds.

The main migratory habits of shorebirds have likely evolved with time constraints being the major selective force (Alerstam and Lindström 1990; Lindström and Alerstam 1992). Notably shorebirds breeding at high northern latitude are thought to be particularly hard‐pressed for time during spring (i.e., northward) migration. A time‐minimization strategy (Hedenström and Alerstam 1997) is essential to enable a timely arrival at the breeding grounds to provide optimal use of the short seasonal peak in food abundance, which is of vital importance to the birds' breeding success.

A typical feature of migratory birds, as well as some other migrant taxa, is the build‐up of fuel stores in preparation for migration. Fuel deposition rate or “fueling rate” is dependent on body mass and importantly determines speed of migration (Lindström 2003) and migration strategies (Gudmundsson et al. 1991; Alerstam 2001). These strategies can be divided according to energy requirements and flight distances into three categories: hop, skip, and jump, from the less energetically demanding strategy to hop short distances, to the energetically challenging strategy of accumulating large stores of extra fuel to jump long distances between sites (Piersma 1987). Whether a migrant adopts a hop, skip, or jump strategy is importantly determined by the difference in quality of sites along the flyway (Piersma 1987). Under the time‐minimization hypothesis, the two extremes, hopping and jumping, will evolve if the potential for fueling is evenly or unequally distributed along a flyway, respectively (Alerstam and Hedenström 1998). The energetic costs associated with carrying extra fuel from particularly good staging sites are rewarded with a faster migration compared to when a hopping strategy had been used (Gudmundsson et al. 1991). The maximum extent of such jumps is ultimately limited by physiological and flight mechanical constraints on fuel accumulation.

Fueling rates show a significant correlation with absolute latitude in red knot *Calidris canutus* worldwide (Piersma et al. 2005) and western sandpiper *Calidris mauri* in America (Williams et al. 2007). On the other hand, Lyons et al. (2008) and Schaub and Jenni (2001) did not find a significant correlation between fueling rates and latitude in semipalmated sandpipers *Calidris pusilla* in North America and four species of passerine birds migrating between Europe and Africa, respectively. Metabolized energy intake is considered the most important determinant of fueling rates (Lindström 2003); therefore, variations in fueling rates between sites may correlate to local differences in food availability (e.g., Schaub and Jenni 2001; Piersma et al. 2005; Van Gils et al. 2005). Poor‐quality feeding sites should be used less intensively and possibly even be skipped during spring migration, depending on the relative quality across sites along the flyway and the distances between them. We suggest that latitudinal patterns in fueling rates, if substantiated, could have considerable repercussions for the migratory strategy of these birds and notably for the distances covered in each migratory leg and overall migration speed.

We predict that for an optimal, time‐minimizing migrant facing latitudinal variation in potential fueling rates: (1) a northward migrant heading to breeding grounds in the Northern Hemisphere from sites in the Southern Hemisphere will use big jumps that will take it to an equivalent or higher latitude in the opposite hemisphere in order to reach high‐quality staging sites and skip low‐quality sites in the tropics; and (2) once having passed the equator, the migrant will use small steps, and fuel sufficiently only to make it to the next suitable staging site (to avoid the energetic cost of carrying excessive amounts of fuel). If these predictions are true, it may be beneficial to spend the nonbreeding season away from the equator at higher latitudes in search of better fueling conditions. Although thus covering a longer distance, this longer journey may result in a higher net speed of migration during the return migration to the breeding grounds.

The aim of this study was to comprehensively investigate (1) the effect of latitude on fueling rates in different migratory shorebirds at a global scale; (2) investigate the potential underlying factors relating to such a pattern with respect to latitude, that is (i) total intertidal biomass in different sites worldwide as an estimation of prey availability and (ii) primary productivity (measured using chlorophyll a concentration); and (3) examine whether our assumptions of a latitudinal pattern in fueling rates lead to the above predicted (1–2) migration strategies using satellite transmitter data and geolocation dataloggers (“geolocator”) data for three high‐latitude breeding shorebird species migrating along the East Asian‐Australasian Flyway.

## Methods

**Figure 1:**
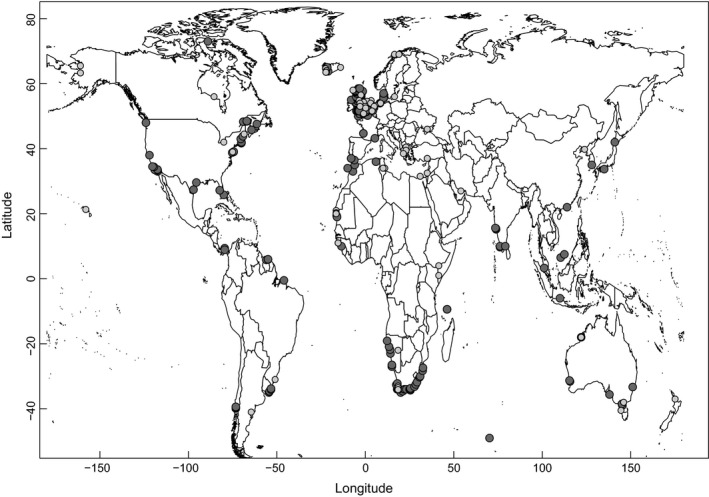
Sampling locations of total intertidal biomass (g AFDM m^−2^; dark gray circles) and sites where fueling rates were measured (mass gain in g day^−1^; light gray circles). All data were obtained from the literature (see text for details).

Fuel deposition rate (“fueling rate”) data (expressed as mass increase in grams per day, g day^−1^) and lean body mass data for migratory shorebirds were collected from the literature for different geographical locations (Zwarts et al. 1990; Gudmundsson et al. 1991; Piersma et al. 2005; Ens et al. 2006; Lindström et al. 2011; Ma et al. 2013) (Fig. 1). The data were based on mass gain within staging shorebird population or from average mass gains in retrapped individuals (Appendix S2).

Published data on total intertidal biomass of benthic macrofauna (g ash free dry mass [g AFDM] m^−2^) (“total biomass”) worldwide were collated (Warwick and Ruswahyuni 1987; Piersma et al. 1993; Masero et al. 1999; Ricciardi and Bourget 1999; Kober and Bairlein 2006; Jing et al. 2007; Purwoko and Wolff 2008; Fig. 1). Only data collected during the periods in which actively migrating shorebirds might have been present were used.

Primary productivity in the areas where data on total biomass and fueling rates were collected (limited to 25 km from the coast line) was downloaded from Ocean Productivity (http://www.science.oregonstate.edu/ocean.productivity/index.php). These primary productivity data were remotely estimated as a function of chlorophyll a, available light, and the photosynthetic efficiency averaged over 8 days. These descriptions are based on the vertically generalized production model (VGPM) (Behrenfeld and Falkowski 1997).

To investigate whether latitudinal variation in potential fueling rates has the predicted effects on migratory strategies, as outlined in the introduction, the sequence of sites used on northward migration for three shorebird species was analyzed. Only stopover sites that were visited for a duration of at least 4 days were considered because by stopping for less than 4 days, the bird is likely to gain only very little mass (Warnock 2010). The location of each stopover site was used to calculate the distance covered in each leg (i.e., distance between each stopover site).

Routes were reconstructed from data downloaded from geolocators deployed and retrieved by the Victorian Wader Study Group in South Australia (for ruddy turnstone *Arenaria interpres* and sanderling *Calidris alba*) and in Victoria and Tasmania (ruddy turnstone). Of the 56 tracks available for ruddy turnstone, five have been published previously, and details on these tracks can be found in Minton et al. (2010). Geolocators are light enough to be fitted on bird species as small as 15 g. However, their accuracy is limited to about 200 km of latitude and 50 km of longitude (Welch and Eveson 1999; Phillips et al. 2004). In our study, birds on northward migration are traveling over a total distance exceeding 13,000 km and refuel at about 3–4 sites along the route. The spatial accuracy of geolocators is therefore sufficient for our specific aim of analyzing the distance traveled between each stopover site.

Although much higher spatial accuracy can be attained with satellite transmitters, their weight prohibits its use in the majority of wader species. We used published routes based on satellite transmitters of two bar‐tailed godwit subspecies big enough for this tracking technique, *Limosa lapponica baueri* and *Limosa lapponica menzbieri* (Battley et al. 2012).

### Statistics

Because we expected a U‐shaped relationship with latitude (i.e., low values around the equator, increasing toward the poles), we used general linear modeling with both latitude and latitude squared as dependent variables (i.e., a second‐order polynomial model) to examine the effect of latitude on fueling rates, primary productivity and total biomass.

Interspecific variation in body mass has been suggested to affect fueling rates (Lindström 2003) and we therefore included lean body mass alongside latitude as predictor variables in our analyses of fueling rates.

Additionally, a phylogenetic mixed model was used to examine the effect of latitude and lean body mass on fueling rates. This phylogenetic mixed model allows random phylogenetic effects to be included. Using R (R Core Team 2014), we fitted the polynomial model using a Bayesian approach by applying the MCMCglmm package. For the phylogeny, a tree based on data provided in Thomas et al. (2004) was applied (Appendix S1). Parameter‐expanded priors were specified following Hadfield (2010).

Prior to analyses, fueling rates, lean body mass, primary productivity, and total biomass were 10‐log transformed to obtain normality in all models. Insignificant predictor variables were pruned from the models.

Reaching the target destination (breeding grounds), the remaining flight distance to that target, becomes progressively shorter. Under the assumption that birds will follow the shortest route to their ultimate target, when plotting leg flight distance against latitude, it was expected that leg flight distances would fall on or below the line depicting the shortest distance between the departure staging site and the ultimate target. For regressions between flight distance and latitude, *H*_*o*_ for the slope is thus not the default zero, but rather half of the slope of the line depicting the maximum distance from any staging site to the ultimate target, as leg flight distances under the null hypothesis will be randomly distributed in between zero and the maximum distance line (Fig. 3).

All statistical analyses were conducted using R version 3.0.3 (R Core Team 2014).

## Results

**Figure 2:**
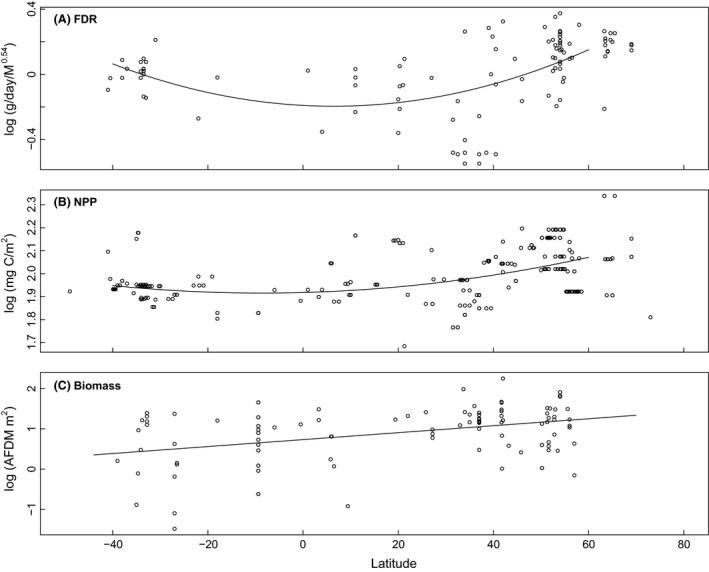
The relationship between latitude and (A) fuel deposition rates (FDR), corrected for lean body mass (M^0.54^), in 13 shorebird species measured as mass increase in g day^−1^, (B) mean net primary productivity (NPP) at the sites where FDR and biomass data were collected, measured as a function of chlorophyll a, available light, and the photosynthetic efficiency, and (C) total intertidal biomass (biomass). All locations of data collection are shown in Figure 1.

Based on general linear modeling, latitude had a significant effect on fueling rates only in its quadratic form (*β *= 0.0001, *P* < 0.01) (Fig. 2A), suggesting a U‐shaped relationship with the minimum located at the equator. In the same model, lean body mass had a significant effect on fueling rates (*β *= 0.56, *P* < 0.01).

Accounting for phylogenetic effects using a phylogenetic mixed model, we found that again fueling rates were positively correlated with latitude only in its quadratic form (slope: *γ *= 0.0002; *P*_MCMC_ < 0.001). Contrastingly, however, body mass had no additional significant effect on fuel deposition rate.

Latitude had a significant effect on primary productivity in the polynomial function (latitude: *β *= 0.00057, *P* < 0.01, latitude^2^: *β *= 0.000033, *P* < 0.01), thus confirming a U‐shaped relationship with latitude (Fig. 2B).

Latitude in its quadratic form had no significant effect on total biomass (*P *=* *0.42), whereas latitude in its linear form had a significant effect (*β *= 0.025, *P *<* *0.01). This implies that a latitudinal trend exists from south to north and not a U‐shaped relationship as we had predicted (Fig. 2C).

**Figure 3:**
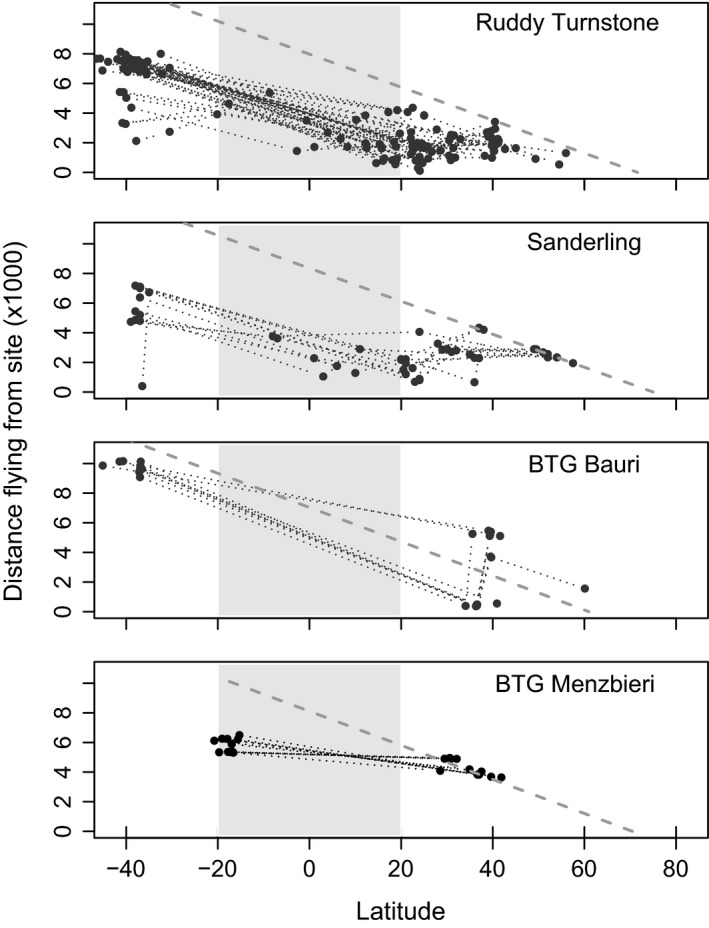
Flight distances (km) for each leg during northward migration. In each panel, the observed distance covered is plotted against the starting staging site latitude for the species: ruddy turnstone, sanderling, bar‐tailed godwit (BTG) from the subspecies *baueri* and *menzbieri*. In each panel, the broken diagonal gray line represents the maximum distance from any staging site to the breeding grounds via a great circle route. Gray‐shaded areas represent low‐latitude regions (i.e., tropical, ranging between −20° and 20° of latitude). The dotted lines connect individual points.

Considering the limitation of H_0_ (see *Methods*), the migration routes from geolocator and satellite transmitter data obtained from trans‐equatorial migrants showed a significant negative relationship between flight distance and latitude during northward migration toward the breeding grounds for the species ruddy turnstone (*β *= −76.3, *F*_1,145_ = 337.1, *P *<* *0.01), sanderling (*β *= −34.6, *F*_1,51_ = 29.6, *P *<* *0.01), bar‐tailed godwit from the subspecies *baueri* (*β* = −88.5, *F*_1,23_ = 67.9, *P *<* *0.01), and *menzbieri* (*β* = −34.3, *F*_1,20_ = 107, *P *<* *0.01) (Fig. 3).

## Discussion

In this study, we showed that migration strategy in four trans‐equatorial migrating shorebirds is characterized by long migration legs (jumps) when leaving staging sites at temperate Southern Hemisphere latitudes, followed by relatively shorter hops/skips toward the final destination, their Arctic breeding grounds. These shorter hops/skips after a major jump may be a result of fine‐tuning timing of arrival at the breeding grounds. However, according to the time‐minimization hypothesis, the long initial jump across the equator followed by shorter hops/skips may also result from a specific pattern in the potential for fuel deposition along the birds' flyway. Using an extended data set on fueling rates and flight distances derived from the literature and from geolocator and satellite transmitters, we confirmed earlier suggestions of a latitudinal trend in fueling rate in shorebirds, where fueling rates decrease from high to low latitudes in the Northern Hemisphere (Piersma et al. 2005; Williams et al. 2007; Lyons et al. 2008), but more importantly, we here show that this latitudinal trend exists for all species in this study in both hemispheres. Given this confirmed latitudinal trend in fueling rates, the typical migration strategies presented here are consistent with the predictions of the time‐minimization hypothesis.

Although we could not confirm a correlation between fueling rate and lean body mass using the phylogenetic model, the effect of body mass on fueling rates has been suggested in earlier studies (e.g., Lindström 2003). Due to corrections for phylogeny, this effect may have been masked in our analysis, as closely related birds tend to be of similar size. The strong effect of body mass on fueling rates using a polynomial model, excluding an effect of phylogeny, supports this inference.

There are a number of potential explanations for why latitude is such an important factor in determining fueling rates. *Firstly*, although here confirmed only from south to north (and not a U‐shaped form), latitudinal trends have been shown in (surface) prey biomass (Piersma et al. 1993; Mathot et al. 2007; Purwoko and Wolff 2008) and now also in primary productivity in this study for both hemispheres. *Secondly*, migrants experience long days for most of the year by crossing the equator. This is notably the case when they are staging at high latitudes during summer, which would consequently allow for increased feeding time (Bauchinger and Klaassen 2005). *Thirdly*, high foraging and feed‐intake rates might be constrained by high ambient temperatures (cf. heat dissipation limitation theory; Speakman and Król (2010); see also Kurnath and Dearing (2013)). Indeed, Battley et al. (2003) found intake rates in waders staging in tropical regions to be limited, for which heat stress was suggested to be an important factor. Low temperatures at high latitudes may assist in heat dissipation, and thus promote higher fuel deposition rates. *Finally*, although explaining the increasing trend in fuel deposition rate in the Northern Hemisphere only, migrants may increase fueling rate at higher latitude because they become increasingly more pressed for time to reach the breeding grounds in time (Reneerkens et al. 2007; Williams et al. 2007).

Data on flight leg distance in relation to the latitude of the departure site (Fig. 3) confirmed our predicted effects of latitudinal variation in fueling rates on migration strategies. These predictions were based on combining the time‐minimization hypothesis and the positive relationship between fueling rate and absolute latitude. Most individual shorebirds start their northward migration from their relatively high‐latitude nonbreeding site, toward the breeding grounds with a jump strategy. This jump is followed by a skipping/hopping strategy toward the breeding ground after passing the equator. Not all individuals follow the shortest distance from their wintering grounds to the breeding grounds. This is evident from the points above the line depicting the distance between the departure staging site and the ultimate target for the two subspecies of bar‐tailed godwit (Fig. 3). These detours from the “shortest‐distance‐to‐goal” may actually result in a higher net speed of migration due to better feeding conditions (Alerstam 2001).

Low fueling rates may not be the only factor stimulating some migrants to avoid sites at low latitudes around the equator. At various locations across the globe, a number of shorebird studies have found disease prevalence to be negatively correlated with absolute latitude (Mendes et al. 2005; Clark et al. unpubl. data). This trend can be explained by the positive correlation between vector activity and within‐vector parasite development with temperature (Santiago‐Alarcon et al. 2012). Moreover, predation pressure may also be higher around the tropics, decreasing with latitude, although so far this trend was substantiated only in high latitudes (McKinnon et al. 2010). These factors may also add to explaining the migratory tracking data obtained and add to the range of factors that may urge migrants (not exclusively waders or birds) to avoid low‐latitude sites.

Many migrants forage more or less continuously while migrating, with food being regularly distributed along their migratory path, reducing the immediate need for the build‐up of migratory stores. Still, also in these cases of continuous food availability but with variations in the amounts of food in space, the time‐minimization hypothesis would expect the skipping or jumping of areas with relatively low food availability (Gudmundsson et al. 1991). For terrestrial migrants, migration range is importantly restricted by ecological barriers, such as oceans and deserts. But given the latitudinal trends in productivity, it might be expected that similar patterns are present in trans‐equatorial migration strategies in other bird groups and marine animal taxa. Indeed, this is also the case in short‐tailed shearwaters (*Ardenna tenuirostris*; Carey et al. 2014) as well as sooty shearwaters (*Puffinus griseus*; Shaffer et al. 2006) which travel south, presumably to exploit highly productive Antarctic water to gain sufficient weight to migrate through the less productive waters of the tropics (Carey et al. 2014). Generally, mass‐specific energetic costs of locomotion decrease with animal size, being lowest in swimmers, followed by flyers and finally animals using terrestrial locomotion (Schmidt‐Nielsen 1972; Hein et al. 2012). From an energetic cost perspective, marine species might therefore also engage in trans‐equatorial migrations driven by latitudinal patterns in food availability. However, many marine species have anti‐tropical distribution patterns (i.e., the same species have separate populations on both sides of the equator). The lower speed of locomotion in swimmers relative to flyers may restrict the distance covered within the annual cycle (Alexander‐McNeill 2002; Johansson et al. 2014).

The latitudinal pattern in the suitability of staging sites for migratory fueling confirmed here may have been of great importance in the evolution of migratory strategies. This is highlighted by the migration itineraries of some shorebird species *en route* from their Southern Hemisphere nonbreeding areas to their Arctic breeding grounds. Therefore, relatively high‐latitude staging sites (further from the tropics) may also be of particular importance for the successful completion of migratory journeys (Aharon‐Rotman et al. 2015). Migratory shorebirds along the East Asian‐Australasian Flyway, which includes the species analyzed in this work, are facing many challenges. The largest disturbances and threats are observed in the staging sites in the Yellow Sea area, located at about 38°N. These sites may therewith currently be acting as the primary ecological bottlenecks for the migratory shorebirds that have relied on them in the past, also explaining their downward population trends (R. Fuller pers. comm.; MacKinnon et al. 2012). The here proposed concept of migratory birds disproportionately relying on high‐latitude staging areas thus needs to be considered when prioritizing conservation efforts.

## Conflict of Interest

None declared.

## Supporting information


**Appendix S1.** Phylogenetic tree used for the phylogenetic mixed model, pruned from Thomas et al. (2004).

**Appendix S2.** A summary of fuel deposition rate data collected from the literature.
