# Supplementary figures and images for: Why fly the extra mile? Latitudinal trend in migratory fuel deposition rate as driver of trans‐equatorial long‐distance migration
Source: Ecol Evol. 2016 Aug 25;6(18):6616–24. doi: 10.1002/ece3.2388 (PMC5058532; doi:10.1002/ece3.2388)

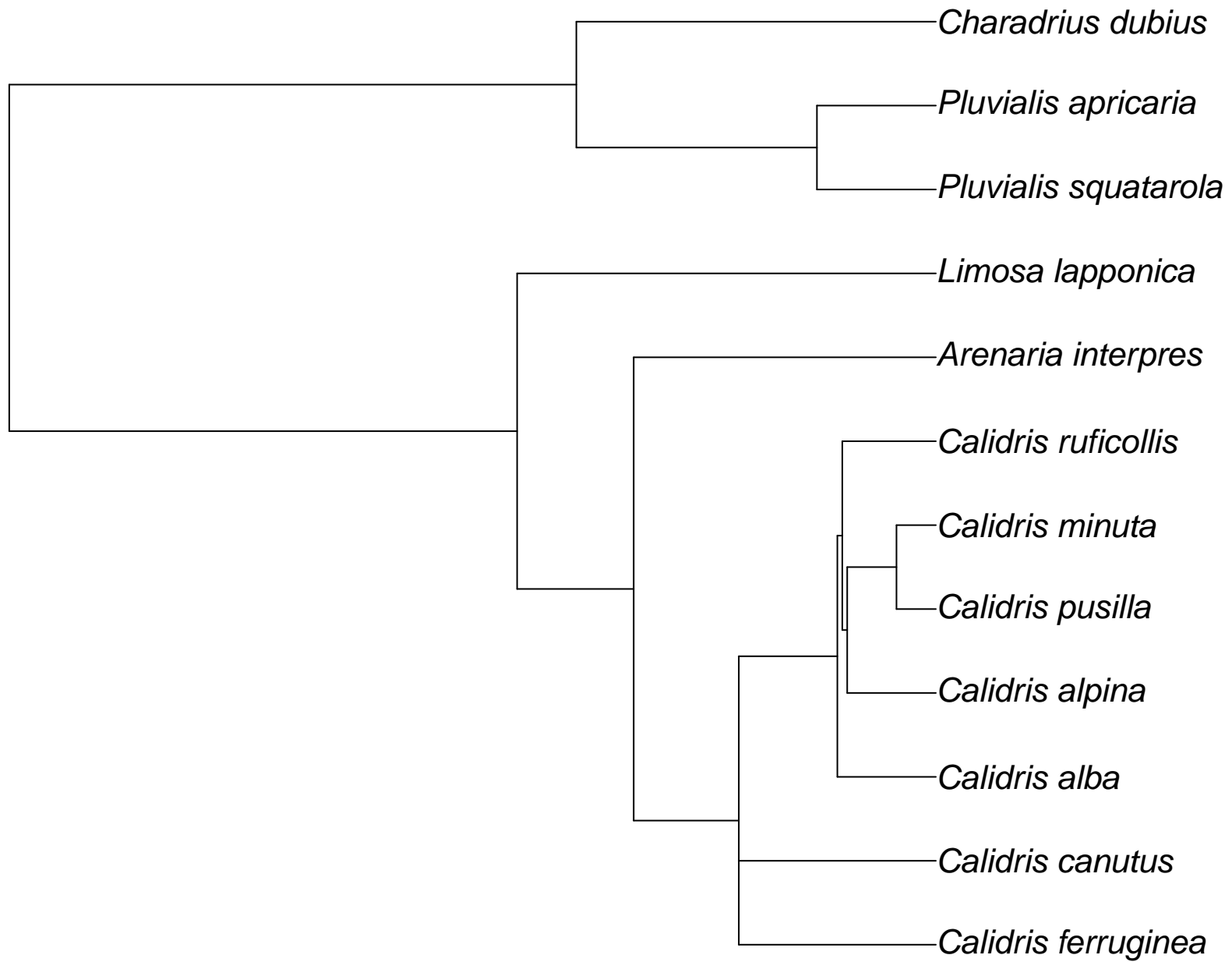

Supplement: Supplementary file 1 — Appendix S1. Phylogenetic tree used for the phylogenetic mixed model, pruned from Thomas et al. (2004). [file ECE3-6-6616-s001.pdf]
