# Supplementary material for: Why fly the extra mile? Latitudinal trend in migratory fuel deposition rate as driver of trans‐equatorial long‐distance migration
Source: Ecol Evol. 2016 Aug 25;6(18):6616–24. doi: 10.1002/ece3.2388 (PMC5058532; doi:10.1002/ece3.2388)
Supplement: Supplementary file 2 — Appendix S2. A summary of fuel deposition rate data collected from the literature. [file ECE3-6-6616-s002.docx]

| Latitude | Longitude | Country/Area | Species | LBM^*^ | FDR^**^(g^-1^) | Month data was collected | Source*** |
| --- | --- | --- | --- | --- | --- | --- | --- |
| -41 | -64.5 | Argentina | Red Knot | 118 | 0.6 | NA | 3 |
| -40.5 | 144.5 | Australia | Red necked Stint | 30 | 0.9 | 3 | 1 |
| -38 | 146 | Australia | Curlew Sandpiper | 53 | 0.9 | 3 | 1 |
| -38 | 146 | Australia | Red Knot | 118 | 1.6 | NA | 3 |
| -37 | 175.1 | New Zealand | Red Knot | 118 | 1.2 | NA | 3 |
| -34.1 | 18.5 | South Africa | Red Knot | 118 | 1.5 | NA | 2 |
| -34.1 | 18.5 | South Africa | Sanderling | 50 | 0.9 | NA | 2 |
| -34.1 | 18.5 | South Africa | Turnstone | 100 | 1.1 | NA | 2 |
| -33.5 | 18.5 | South Africa | Curlew Sandpiper | 48 | 0.52 | 3 | 4 |
| -33.5 | 18.5 | South Africa | Curlew Sandpiper | 53 | 1 | 3 | 1 |
| -33.5 | 18.5 | South Africa | Grey plover | 200 | 1.2 | 4 | 1 |
| -33.5 | 18.5 | South Africa | Little stint | 21 | 1.5 | 4 | 1 |
| -33.5 | 18.5 | South Africa | Red Knot | 118 | 1.2 | 4 | 1 |
| -33.5 | 18.5 | South Africa | Sanderling | 54 | 1.1 | 4 | 1 |
| -33.5 | 18.5 | South Africa | Turnstone | 90 | 1 | 4 | 1 |
| -33 | 18.5 | South Africa | Red Knot | 118 | 1.5 | NA | 3 |
| -33 | 18.5 | South Africa | Sanderling | 50 | 0.5 | 4 | 4 |
| -31 | -51 | Brazil | Red Knot | 118 | 3.1 | NA | 3 |
| -22 | 18.5 | Namibia | Little stint | 21 | 0.32 | 4 | 4 |
| -18 | 122 | Australia | Red Knot | 118 | 0.9 | NA | 3 |
| 1 | 41.5 | Kenya | Little stint | 20 | 1.1 | 4 | 1 |
| 4 | 41.5 | Kenya | Little stint | 20 | 0.23 | NA | 4 |
| 11 | -15.4 | Guinea Bissau | Curlew Sandpiper | 48 | 0.33 | 4 | 4 |
| 11 | -15.4 | Guinea Bissau | Grey plover | 230 | 1.2 | NA | 4 |
| 11 | -15.4 | Guinea Bissau | Red Knot | 118 | 0.9 | NA | 3 |
| 11 | -15.4 | Guinea Bissau | Turnstone | 105 | 0.7 | NA | 4 |
| 20 | -16.5 | Mauritania | Curlew Sandpiper | 48 | 0.48 | 4 | 4 |
| 20 | -16.5 | Mauritania | Little stint | 21 | 0.22 | NA | 4 |
| 20.3 | -16.5 | Mauritania | Curlew Sandpiper | 53 | 0.7 | 10 | 1 |
| 20.3 | -16.5 | Mauritania | Sanderling | 50 | 0.36 | 4 | 4 |
| 20.3 | -16.5 | Mauritania | Turnstone | 105 | 1.3 | 4 | 1 |
| 21 | -16.5 | Mauritania | Red Knot | 118 | 0.7 | NA | 3 |
| 21.3 | -158 | Hawaii | Golden plover | 190 | 1.7 | 4 | 1 |
| 27 | 54 | Persian Gulf UAE | Curlew Sandpiper | 48 | 0.9 | 5 | 4 |
| 31.5 | 31 | Egypt | Curlew Sandpiper | 48 | 0.1 | NA | 4 |
| 31.5 | 31 | Egypt | Little stint | 21 | 0.31 | 4 | 4 |
| 32.5 | 35 | Israel | Dunlin | 42 | 0.1 | NA | 4 |
| 32.5 | 35 | Israel | Little stint | 21 | 0.5 | NA | 4 |
| 34 | 10 | Tunisia | Curlew Sandpiper | 48 | 0.1 | NA | 4 |
| 34 | 11 | Tunisia | Dunlin | 42 | 0.15 | 4 | 4 |
| 34 | 10 | Tunisia | Little stint | 21 | 0.1 | 4 | 4 |
| 34 | 10 | Tunisia | Turnstone | 105 | 4 | NA | 4 |
| 37 | 35 | Turkey | Dunlin | 42 | 0.1 | NA | 4 |
| 37 | 35 | Turkey | Little stint | 21 | 0.1 | NA | 4 |
| 37 | 35 | Turkey | Little stint | 21 | 0.34 | NA | 4 |
| 38.5 | 21.5 | Greece | Curlew Sandpiper | 48 | 0.1 | NA | 4 |
| 39 | -75 | USA | Red Knot | 118 | 4.6 | NA | 3 |
| 39.5 | 23 | Greece | Curlew Sandpiper | 48 | 1 | NA | 4 |
| 39.8 | 123.9 | China | Great knot | 144 | 3.55 | 5 | 6 |
| 40.5 | 23 | Greece | Dunlin | 42 | 0.1 | 4 | 4 |
| 40.5 | 23 | Greece | Grey plover | 230 | 0.7 | 4 | 4 |
| 40.5 | 23 | Greece | Little stint | 20 | 1.9 | 4 | 4 |
| 42 | -80 | USA | Semipalmated sandpiper | 23 | 4 | 8 | 1 |
| 44.5 | -68.5 | USA | Semipalmated sandpiper | 23 | 1.5 | 8 | 1 |
| 46 | 35 | Black Sea | Dunlin | 42 | 0.87 | NA | 4 |
| 46 | 35 | Black Sea | Little stint | 21 | 0.5 | NA | 4 |
| 50.8 | -1 | England | Dunlin | 42 | 3.9 | 8 | 1 |
| 51.6 | 3.8 | The Netherlands | Turnstone | 105 | 0.5 | 4 | 4 |
| 51.6 | 3.8 | The Netherlands | Turnstone | 105 | 2.9 | 5 | 4 |
| 52.5 | 0.2 | England | Dunlin | 40 | 1.1 | 5 | 1 |
| 52.5 | 0.2 | England | Grey plover | 200 | 1.8 | 5 | 1 |
| 52.5 | 0.2 | England | Grey plover | 230 | 3.37 | 5 | 4 |
| 53 | 5 | Wadden Sea | Bar tailed Godwits (M) | 240 | 2.8 | 5 | 1 |
| 53 | -3 | England | Curlew Sandpiper | 48 | 5.4 | 9 | 1 |
| 53 | 0 | England | Red Knot | 118 | 1.7 | NA | 3 |
| 53 | -3 | England | Ringed plover | 49 | 1.2 | 5 | 1 |
| 53.25 | 5.5 | Wadden Sea | Dunlin | 42 | 2.1 | 5 | 1 |
| 53.25 | 5.5 | Wadden Sea | Dunlin | 42 | 0.4 | 4 | 1 |
| 54 | 8.5 | Wadden Sea | Bar tailed Godwits (F) | 260 | 4.5 | 5 | 1 |
| 54 | 8.5 | Wadden Sea | Bar tailed Godwits (F) | 260 | 0.4 | 4 | 1 |
| 54 | -3 | England | Dunlin | 42 | 2.3 | 5 | 1 |
| 54 | 8.5 | Wadden Sea | Grey plover | 230 | 4.6 | 5 | 1 |
| 54 | 8.5 | Wadden Sea | Grey plover | 230 | 8.6 | 5 | 4 |
| 54 | -3 | England | Red Knot | 118 | 1.2 | 4 | 1 |
| 54 | 8 | Germany | Red Knot | 118 | 2.8 | NA | 3 |
| 54 | 8 | Germany | Red Knot | 118 | 3 | NA | 3 |
| 54 | 8.6 | Germany | Red Knot | 118 | 2.8 | NA | 2 |
| 54 | 8.6 | Germany | Red Knot | 118 | 3 | NA | 2 |
| 54 | 8.5 | Wadden Sea | Red Knot | 118 | 2.2 | 4 | 1 |
| 54 | 8.5 | Wadden Sea | Red Knot | 96 | 3.6 | 5 | 1 |
| 54 | -3 | England | Ringed plover | 49 | 3 | 5 | 1 |
| 54 | -3 | England | Turnstone | 90 | 1.5 | 5 | 1 |
| 54 | 3 | England | Turnstone | 105 | 1.4 | NA | 2 |
| 54.3 | -1 | England | Sanderling | 54 | 2.1 | 5 | 1 |
| 54.6 | 1 | England | Sanderling | 50 | 0.8 | NA | 2 |
| 54.8 | 3.3 | England | Sanderling | 50 | 0.9 | NA | 2 |
| 54.8 | -3.5 | Scotland | Sanderling | 50 | 1.9 | 5 | 1 |
| 56 | 16.5 | Sweden | Dunlin | 42 | 2.4 | 9 | 1 |
| 56 | -85.5 | Canada | Semipalmated Sandpiper | 23 | 1.5 | 7 | 1 |
| 56.5 | -2.5 | Scotland | Turnstone | 90 | 1.7 | 5 | 1 |
| 58 | -7 | Scotland | Red Knot | 96 | 4.9 | 8 | 1 |
| 63.3 | -161 | Alaska | Sharp-tailed sandpiper | 61.5 | 0.35 | 9 | 5 |
| 63.3 | -161 | Alaska | Sharp-tailed sandpiper | 61.5 | 3.7 | 9 | 5 |
| 63.5 | -22.3 | Iceland | Red Knot | 118 | 3 | NA | 2 |
| 63.5 | -22 | Iceland | Red Knot | 118 | 2.6 | NA | 2 |
| 63.5 | -22.3 | Iceland | Sanderling | 50 | 1.7 | NA | 2 |
| 63.5 | -22.3 | Iceland | Turnstone | 105 | 3.2 | NA | 2 |
| 64 | -22 | Iceland | Red Knot | 118 | 2.1 | 5 | 1 |
| 64 | -22 | Iceland | Turnstone | 90 | 2.1 | 5 | 1 |
| 64.6 | -22.5 | Iceland | Red Knot | 118 | 3.1 | NA | 2 |
| 64.6 | -22.5 | Iceland | Turnstone | 105 | 3.8 | NA | 2 |
| 65 | -14 | Iceland | Red Knot | 118 | 2.9 | NA | 3 |
| 65.5 | -161 | Alaska | Turnstone | 90 | 3.7 | 9 | 1 |
| 69 | 16.5 | Norway | Red Knot | 118 | 2.2 | 5 | 1 |
| 69 | 16.5 | Norway | Red Knot | 118 | 2.6 | NA | 2 |
| 69 | 19 | Norway | Red Knot | 118 | 2.7 | NA | 3 |

^*^lean body mass

^**^fuel deposition rate

^***^ ^1^ Zwarts *et al*. 1990, ^2^ Gudmundsson *et al*. 1991, ^3^ Piersma *et al*. 2005, ^4^ Ens *et al.* 2006, ^5^Lindström *et al.* 2011, ^6^ Ma *et al*. 2013
